# Oxidation-Triggerable Liposome Incorporating Poly(Hydroxyethyl Acrylate-*co*-Allyl methyl sulfide) as an Anticancer Carrier of Doxorubicin

**DOI:** 10.3390/cancers12010180

**Published:** 2020-01-10

**Authors:** Jin Ah Kim, Dong Youl Yoon, Jin-Chul Kim

**Affiliations:** Department of Medical Biomaterials Engineering, College of Biomedical Science and Institute of Bioscience and Biotechnology, Kangwon National University, 192-1, Hyoja 2 dong, Chuncheon, Kangwon-do 200-701, Korea

**Keywords:** dioleoylphosphatidylethanolamine, liposomes, poly(hydroxyethyl acrylate-co-allyl methyl sulfide) copolymer, folate, oxidation-sensitive release, doxorubicin, cellular interaction, in vitro anti-cancer activity

## Abstract

Since cancer cells are oxidative in nature, anti-cancer agents can be delivered to cancer cells specifically without causing severe normal cell toxicity if the drug carriers are designed to be sensitive to the intrinsic characteristic. Oxidation-sensitive liposomes were developed by stabilizing dioleoylphosphatidyl ethanolamine (DOPE) bilayers with folate-conjugated poly(hydroxyethyl acrylate-co-allyl methyl sulfide) (F-P(HEA-AMS)). The copolymer, synthesized by a free radical polymerization, was surface-active but lost its surface activity after AMS unit was oxidized by H_2_O_2_ treatment. The liposomes with F-P(HEA-AMS) were sensitive to H_2_O_2_ concentration (0%, 0.5%, 1.0%, and 2.0%) in terms of release, possibly because the copolymer lost its surface activity and its bilayer-stabilizing ability upon oxidation. Fluorescence-activated cell sorting (FACS) and confocal laser scanning microscopy (CLSM) revealed that doxorubicin (DOX)-loaded liposomes stabilized with folate-conjugated copolymers markedly promoted the transport of the anti-cancer drug to cancer cells. This was possible because the liposomes were readily translocated into the cancer cells via receptor-mediated endocytosis. This liposome would be applicable to the delivery carrier of anticancer drugs.

## 1. Introduction

Phospholipids are amphiphilic molecules and they were self-assembled in aqueous phase by an entropy-driven process [1,2,3,4,5]. Phosphatidylcholine (PC) is cylindrical in shape and its packing parameter is about 1, so it can be assembled into bilayer vesicles (i.e., liposome). Phosphatidylethanolamine (PE) is conical and its packing parameter is larger than 1, thus it can constitute non-bilayer assemblies (i.e., reversed hexagonal phase). If amphiphilic complementary molecules are inserted in between the head groups of PE molecules, liposomes can be formed [6,7,8]. Hydrophobically modified poly(*N*-isopropylacrylamide) was adopted as a stabilizer for the formation of dioleoylphophatidylethanolamine (DOPE) liposomes [9,10]. If the liposomes encounter a temperature higher than the phase transition temperature of the polymer, the polymer chains can contract and the bilayer domain they can cover and stabilize would decrease, leading to the disintegration of liposomes and the thermally triggered release of its payload. Cholesteryl hemisuccinate (CHEMS) was used as a complementary molecule for the stabilization of DOPE liposomes [11,12,13]. If the liposome suspension is acidified, the head group of CHEMS (i.e., carboxyl group) becomes unionized, its effective size decreases, thus the complementary molecule can lose its ability to stabilize DOPE bilayers, resulting in acidification-triggered release. Hydrophobically modified immunoglobulin G (HmIgG) could stabilize DOPE bilayer and it was claimed to be used as a targetable drug carrier [14]. If the liposomes come in contact with their target site, the HmIgG molecules are supposed to diffuse on the liposomal surface toward antigens and cause the disintegration of the liposomes. Glucose oxidase was immobilized on the surface of the DOPE liposome stabilized with CHEMS to render the liposome sensitive to glucose [15]. The enzyme can convert glucose to gluconic acid and acidify the medium, triggering the release from the pH-sensitive liposomes.

**Figure 1:**
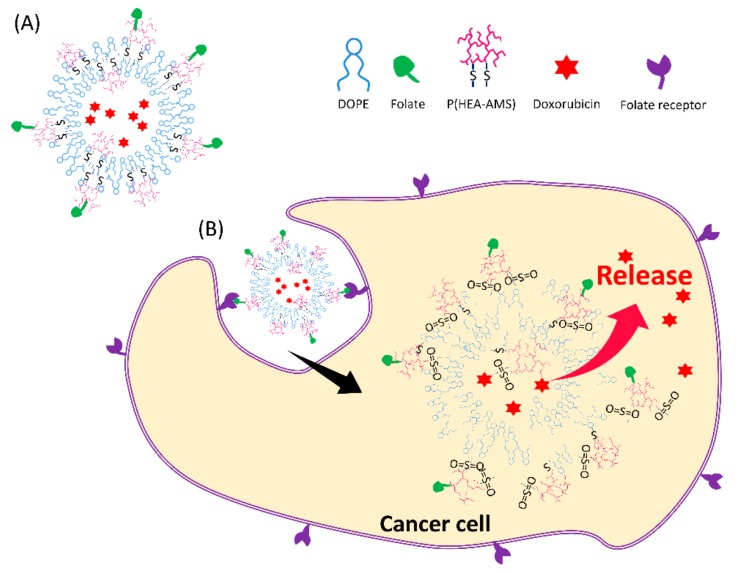
Scheme of oxidation-sensitive dioleoylphophatidylethanolamine (DOPE) liposomes stabilized with liposome incorporating folate-conjugated poly(hydroxyethyl acrylate-co-allyl methyl sulfide) (F-P(HEA-AMS)). AMS can act as a hydrophobic anchor and be hydrophobically intercalated into the bilayers with HEA segments orienting toward aqueous bulk phase. Thus, the copolymer would be able to stabilize DOPE liposomes (**A**). If the liposomes are exposed to an oxidative condition, the methyl sulfide of AMS can be oxidized to methyl sulfone and it would hardly act as a hydrophobic anchor. As a result, the polymeric stabilizer would be desorbed from the liposomal membrane and it would cause the disintegration of liposomes and trigger the release from the liposomes (**B**).

In this study, oxidation-sensitive and cancer cell-targetable liposomes were prepared by stabilizing DOPE liposomes with folate-conjugated poly(hydroxyl ethyl acrylate-co-allyl methyl sulfide) (F-P(HEA-AMS)). Since HEA is hydrophilic and AMS is lipophilic, P(HEA-AMS) is a kind of amphiphilic polymer. AMS can act as a hydrophobic anchor and be hydrophobically intercalated into the bilayers with HEA segments orienting toward aqueous bulk phase. Thus, the copolymer would be able to stabilize DOPE liposomes (Figure 1A). Owing to folate conjugated to the copolymer, the liposomes can target cancer cells that mostly are known to overexpress folate receptors and they would be readily internalized into the cells via receptor mediated endocytosis. Doxorubicin (DOX) was loaded in the liposomes as an anti-cancer drug. DOX is known to kill cancer cells by being inserted between DNA double strands and suppressing the biosynthesis of proteins. It is also known that oxygen free radicals are formed by DOX within cancer cells and they peroxidize the cellular membrane lipids and exterminate the cancer cells [16]. In addition to inherent intracellular ROS, DOX-induced oxygen free radicals would also cause the methyl sulfide of AMS of the copolymer to be oxidized. Once oxidized within cells, AMS would hardly act as a hydrophobic anchor. As a result, the polymeric stabilizer would be desorbed from the liposomal membrane and it would cause the disintegration of liposomes and trigger the release of DOX from the liposomes (Figure 1B).

## 2. Results and Discussion

### 2.1. ^1^H NMR Spectroscopy

**Figure 2:**
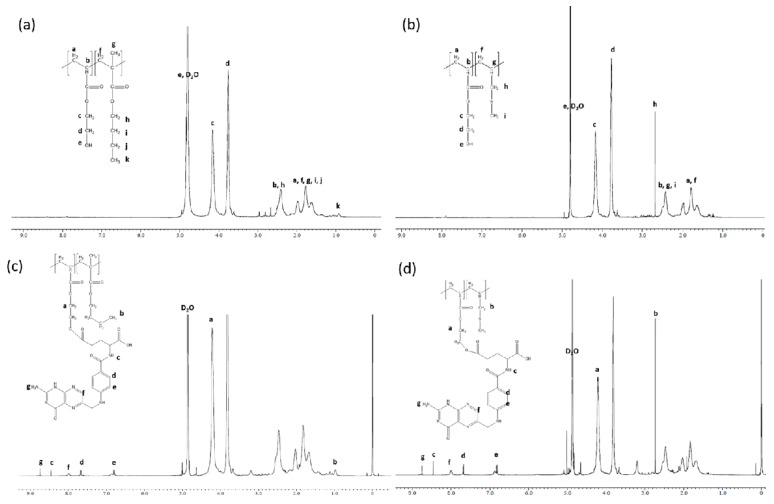
^1^H NMR spectrum of P(HEA-BMA) (**a**) and P(HEA-AMS) (**b**), F-P(HEA-BMA) (**c**) and F-P(HEA-AMS) (**d**).

The ^1^H NMR spectrum of poly(hydroxyethyl acrylate-co-butyl methacrylate) (P(HEA-BMA)), poly(hydroxyethyl acrylate-co-allyl methyl sulfide) (P(HEA-AMS)), folate-conjugated poly(hydroxyethyl acrylate-co-butyl methacrylate) (F-P(HEA-BMA)) and folate-conjugated poly(hydroxyethyl acrylate-co-allyl methyl sulfide) (F-P(HEA-AMS)) are shown in Figure 2. In the ^1^H NMR spectrum of P(HEA-BMA), the methyl group of BMA was found in the range of 0.92–1.06 ppm, two methylene groups next to the methyl group in the range of 1.35–2.14 ppm, the methylene group next to ester bond at 2.41 ppm, the methyl group next to quaternary carbon in the range of 1.35–2.14 ppm, the vinyl methylene group in the range of 1.35–2.14 ppm, the hydroxyl group of HEA at 4.80 ppm, the methylene group next to hydroxyl group at 3.76 ppm, the methylene group next to ester bond at 4.16 ppm, the methine group around 2.41 ppm, and the vinyl methylene group in the range of 1.35–2.14 ppm. The signal of the methyl group of BMA did not overlap with any other signals and the signal of the methylene group next to the hydroxyl group of HEA did not either. Using the area of those signals, the molar ratio of HEA to BMA was calculated to be 100 ÷ 3.5. In the ^1^H NMR spectrum of P(HEA-AMS), the methyl group of AMS was found in the range of 1.63–1.99 ppm, the methylene group next to the sulfur at 2.69 ppm, and the methine group at 2.42 ppm. The signals of HEA of P(HEA-AMS) appeared at almost the same position as those of HEA of P(HEA-BMA). The signal of the methylene group of AMS did not overlap with any other signals and the signal of the methylene group next to the hydroxyl group of HEA did not either. Using the area of those signals, the molar ratio of HEA to AMS was calculated to be 100 ÷ 5.2. The aromatic protons of folate were found at 6.78 ppm, 7.66 ppm and 7.99 ppm, and the units (i.e., HEA, BMA, and AMS unit) of folate-conjugated copolymers were found at almost the same position as those of folate-free copolymers. In the spectrum of F-P(HEA-BMA), the area of the aromatic ring signals was 2.0, and that of the ethyl group signals of HEA unit was 56.9. Thus, the molar ratio of folate to HEA unit was calculated to be about 1 ÷ 14, indicating that folate was conjugated to every 14 HEA units. Since the molar ratio of HEA to BMA was 100 ÷ 3.5, the molar ratio of folate:HEA:BMA of F-P(HEA-BMA) was calculated to be 6.7 ÷ 93.3 ÷ 3.5. In the spectrum of F-P(HEA-AMS), the area of the aromatic ring signals was 3.6, and that of the ethyl group signals of HEA unit was 109.8. Therefore, the molar ratio of folate to HEA unit was calculated to be about 1 ÷ 15. Since the molar ratio of HEA to AMS was 100 ÷ 5.2, the molar ratio of folate/HEA/AMS of F-P(HEA-AMS) was calculated to be 6.2 ÷ 93.8 ÷ 5.2.

### 2.2. Examination of Oxidation of Copolymers by XPS

**Figure 3:**
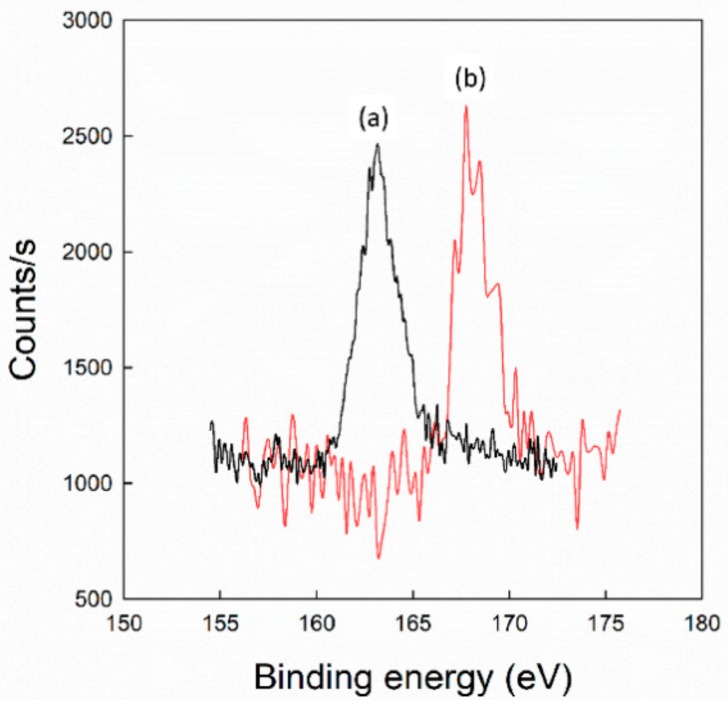
XPS spectrum of P(HEA-AMS) (**a**) and H_2_O_2_-P(HEA-AMS) (**b**).

Figure 3 shows the XPS spectrum of P(HEA-AMS) and H_2_O_2_-P(HEA-AMS). A strong peak around 163.5 eV was found in the spectrum of P(HEA-AMS) and it could be ascribed to the sulfur signal of the sulfide moiety (i.e., the AMS unit). H_2_O_2_-P(HEA-AMS) showed a relatively broad peak centered at 168 eV with a shoulder at 166 eV. Those signals could be assigned to the sulfur electron of the sulfone moiety and that of the sulfoxide one, respectively. Thus, it could be said that the AMS unit was readily oxidized by H_2_O_2_ under our experimental condition.

### 2.3. Measurement of Interfacial Tension

**Figure 4:**
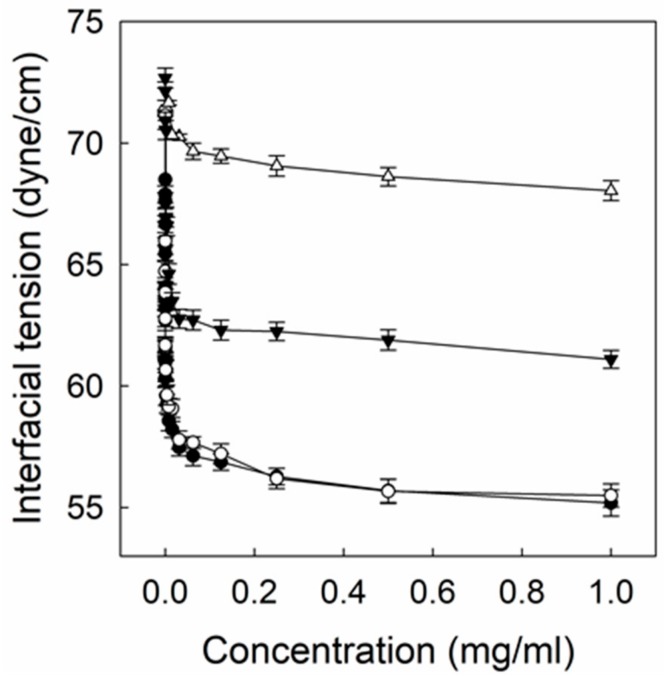
Air/water interfacial tension of P(HEA-BMA) (●), H_2_O_2_-P(HEA-BMA) (○), P(HEA-AMS) (▼), and H_2_O_2_-P(HEA-AMS) solution (△).

Figure 4 shows the air/water interfacial tension of P(HEA-BMA), H_2_O_2_-P(HEA-BMA), P(HEA-AMS), and H_2_O_2_-P(HEA-AMS) solution. The interfacial tension of P(HEA-BMA) solution decreased steeply from 73 dyne/cm to 57 dyne/cm with an increasing concentration in the range of 0–0.02 mg/mL, it decreased gradually from 57 dyne/cm to 56 dyne/cm in the range of 0.02–0.5 mg/mL, and no more decrease was observed in the remaining concentration range. An L type of interfacial tension profile is typical of a surface-active agent [17,18]. The concentration where two tangential lines intersect each other can be assumed to be the critical micellization concentration (CMC) [18,19,20]. The CMC was estimated to be 0.0156 mg/mL. BMA is a hydrophobic monomer and its copolymerization with a hydrophilic monomer (i.e., HEA) would result in the formation of an amphiphilic and interface-active copolymer. Owing to its amphiphilicity, the copolymer chains would be aligned at air/water interface with HEA segments being in water and BMA residue facing air to reduce the interfacial tension. The interfacial tension profile of H_2_O_2_-P(HEA-BMA) solution was almost the same as that of P(HEA-BMA) solution. This suggested that the interfacial activity of P(HEA-BMA) was little changed after treated with H_2_O_2_. In fact, P(HEA-BMA) had no oxidizable groups and its chemical structure would hardly be affected by the oxidizing agent treatment. P(HEA-AMS) solution also exhibited an L type of interfacial tension profile. The plateau interfacial value was about 61 dyne/cm and it was higher than that of P(HEA-BMA), indicating that P(HEA-AMS) was less interface-active than P(HEA-BMA). The ^1^H NMR spectroscopy revealed that the molar content of the hydrophobic monomer (i.e., AMS) of the former copolymer was about 4.9% and it was more than that of the hydrophobic monomer (i.e., BMA) of the latter copolymer, 3.4%. Nevertheless, P(HEA-AMS) was less interface-active. AMS has methylene methyl sulfide (CH_3_-S-CH_2_-) on the side of vinyl group and BMA has butoxy carbonyl group (CH_3_-(CH_2_)_3_-O-C=O-) and methyl group on the side. Thus, AMS seemed to be less hydrophobic than BMA and to be less effective in causing the copolymer to be amphiphilic. This could explain why the interfacial activity of P(HEA-AMS) was lower than that of P(HEA-BMA). The CMC of P(HEA-AMS), estimated by the crossing point of two tangential lines, was about 0.025 mg/mL, and it was higher than that of P(HEA-BMA), about 0.0156 mg/mL. This also indicated that P(HEA-AMS) was less surface-active and less amphiphilic. The interfacial tension of H_2_O_2_-P(HEA-AMS) solution slightly decreased to 68.0 dyne/cm when the concentration increased to 1.0 mg/mL, and it was higher than that of P(HEA-AMS) solution in the full concentration tested. Since a saturation type of decrease pattern in the interfacial tension disappeared and the interfacial tension was reduced only by 4 dyne/cm when increased up to the maximum concentration, it was concluded that P(HEA-AMS) lost most of its interfacial activity after treated with H_2_O_2_. XPS revealed that the sulfide of AMS unit was oxidized to the sulfoxide and the sulfone upon the oxidizing agent treatment (Figure 3). Once P(HEA-AMS) was oxidized, the copolymer would lose its amphiphilicity and its interfacial activity because AMS unit became hydrophilic.

### 2.4. Characterization of DOPE Liposomes Stabilized with Copolymers

**Figure 5:**
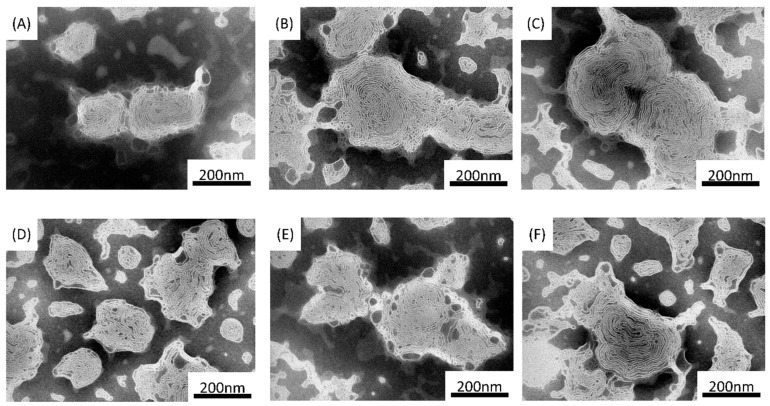
TEM micrograph of liposome/F-P(HEA-BMA)(200/1) (**A**), liposome/F-P(HEA-BMA)(100/1) (**B**), liposome/F-P(HEA-BMA)(50/1) (**C**), liposome/ F-P(HEA-AMS)(200/1) (**D**), liposome/F-P(HEA-AMS)(100/1) (**E**), and liposome/F-P(HEA-AMS)(50/1) (**F**). The magnification was 80,000 times, and bar on each micrograph corresponds to 200 nm.

Figure 5 shows the TEM photo of liposome/F-P(HEA-BMA)(200/1), liposome/F-P(HEA-BMA)(100/1), liposome/F-P(HEA-BMA)(50/1), liposome/F-P(HEA-AMS)(200/1), liposome/F-P(HEA-AMS)(100/1), and liposome/F-P(HEA-AMS)(50/1) that 200/1, 100/1 and 50/1 refer to dioleoylphophatidylethanolamine (DOPE)/copolymer mass ratio. The liposomes were multi-lamellar vesicles regardless of whether the stabilizer was F-P(HEA-BMA) or F-P(HEA-AMS). DOPE is conical because the head (i.e., ethanolamine) is much smaller than the tail (i.e., dioleoyl group) and its packing parameter is more than 1. When dispersed in aqueous solution, DOPE molecules are assembled into reversed hexagonal phase [21,22]. An amphiphilic molecule having a large hydrophilic head and a small hydrophobic tail can be used as a complementary molecule to have DOPE molecules assembled into liposomal bilayers [23,24]. The hydrophobic tail of complementary molecule is likely to be inserted in between the tails of DOPE molecules through hydrophobic interaction while the hydrophilic head would fill the space between the heads of DOPE molecules. F-P(HEA-BMA) and F-P(HEA-AMS) were thought to act as a complementary molecule to stabilize the DOPE liposomal bilayers. The HEA segments of the copolymers would be able to fill the space between the heads of DOPE molecules while the hydrophobic anchors (i.e., BMA and AMS) being anchored in between the tails. This would be a mechanism by which the copolymers could have DOPE molecules assembled into the liposomal bilayers. The quenching % of calcein enveloped in liposomes decreased with increasing the amount of the copolymers. For example, the quenching % of calcein enveloped in liposome/F-P(HEA-BMA)(200/1), liposome/F-P(HEA-BMA)(100/1), and liposome/F-P(HEA-BMA)(50/1) were 55.6%, 51.0%, and 46.2%, respectively. The quenching % of calcein enveloped in liposome/F-P(HEA-AMS)(200/1), liposome/F-P(HEA-AMS)(100/1), and liposome/F-P(HEA-AMS)(50/1) were 57.7%, 55.6%, and 53.9%, respectively.

The specific loading percentages of doxorubicin (DOX) loaded in liposome/F-P(HEA-BMA)(200/1), liposome/F-P(HEA-BMA)(100/1), liposome/F-P(HEA-BMA)(50/1) were 1.82%, 2.35%, and 2.39%, respectively. It seemed that the specific loading % was greater at a lower mass ratio of DOPE to copolymer. This was possibly because the specific loading % of a water-soluble drug is proportional to the size of liposome [25,26]. In fact, the mean hydrodynamic diameter of the liposomes was larger when the DOPE to copolymer mass ratio was lower. The specific loading percentages of DOX loaded in liposome/F-P(HEA-AMS)(200/1), liposome/F-P(HEA-AMS)(100/1), and liposome/F-P(HEA-AMS)(50/1) were 1.85%, 1.98%, 2.21%, respectively. The same reason would be applicable in explaining why the specific loading % was greater at a lower mass ratio of DOPE to copolymer.

The mean diameter of liposomes increased with increasing the amount of the copolymers. For example, the hydrodynamic mean diameter of liposome/F-P(HEA-BMA)(200/1), liposome/F-P(HEA-BMA)(100/1), and liposome/F-P(HEA-BMA)(50/1) were about 163 nm, 273 nm, and 283 nm, respectively. The hydrodynamic mean diameter of liposome/F-P(HEA-AMS)(200/1), liposome/F-P(HEA-AMS)(100/1), and liposome/F-P(HEA-AMS)(50/1) were about 169 nm, 176 nm, and 262 nm, respectively. If the amount of copolymers increases, the amount of DOPE bilayers formed would increase because the copolymers could stabilize DOPE bilayer. An increase in the amount of bilayer would lead to increase in the number of liposomal particle or increase in the number of bilayer per one liposomal particle. Since the mean diameter was proportional to the amount of copolymers, the latter case was likely to take place more favorably than the former case. In fact, as the amount of copolymers was higher, the diameter seemed to be larger and the number of bilayer per one liposomal particle seemed to be greater (Figure 5).

### 2.5. Observation of Oxidation-Sensitive Release

The H_2_O_2_ concentration-dependent release profiles of calcein enveloped in liposome/F-P(HEA-BMA)(200/1), liposome/F-P(HEA-BMA)(100/1), and liposome/F-P(HEA-BMA)(50/1) were presented in Figure S1. The release % of the fluorescence dye enveloped in liposome/F-P(HEA-BMA)(200/1) did not increase markedly with time lapse regardless of what the H_2_O_2_ concentration was and it did not strongly depend on H_2_O_2_ concentration either. For example, the maximum release % obtained when H_2_O_2_ concentration was 0%, 0.5%, 1.0%, and 2.0% was about 0.2%, 3.4%, 5.8%, and 7.8%, respectively. The small increase in the release % with increasing H_2_O_2_ concentration was probably because of the H_2_O_2_-caused oxidation of DOPE molecules. Two double bonds are in the tail of the phospholipid and they are subjected to oxidation [27,28]. Upon the H_2_O_2_-caused oxidation, the double bond of DOPE can be broken down and the hydrophobic chain (i.e., oleoyl group) can be shortened, resulting in the destabilization of DOPE bilayers and the release of the dye. F-P(HEA-BMA) had no oxidation-sensitive groups and it would hardly affect the H_2_O_2_ concentration-dependent release. The H_2_O_2_ concentration-dependent release profile and the release percentages of calcein enveloped in liposome/F-P(HEA-BMA)(100/1) and liposome/F-P(HEA-BMA)(50/1) were almost the same as those of the dye enveloped in liposome/F-P(HEA-BMA)(200/1). This implied that the copolymer had little effect on the H_2_O_2_ concentration-dependent release. In fact, there were no oxidation-sensitive groups in the copolymer.

**Figure 6:**
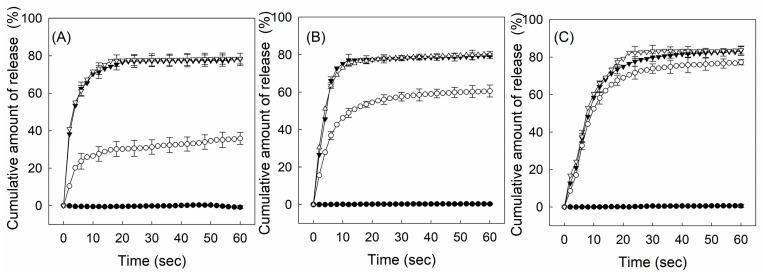
H_2_O_2_ concentration-dependent release profiles of calcein enveloped in liposome/P(HEA-AMS)(200/1) (**A**), liposome/P(HEA-AMS)(100/1) (**B**), and liposome/ P(HEA-AMS)(50/1) (**C**) at 0 % (●), 0.5 % (○), 1.0 % (▼), and 2.0 % (▽) H_2_O_2_ concentration.

Figure 6 shows the H_2_O_2_ concentration-dependent release profiles of calcein enveloped in liposome/F-P(HEA-AMS)(200/1), liposome/F-P(HEA-AMS)(100/1), and liposome/F-P(HEA-AMS)(50/1). Liposome/F-P(HEA-AMS)(200/1) showed no appreciable release during the whole period of release experiment (i.e., 60 s) when no H_2_O_2_ was contained in the release medium, indicating that the liposomes were stable in the buffer solution free of the oxidizing agent. When the concentration of oxidizing agent was 0.5%, the liposomes released their content rapidly for the first few seconds and slowly during the remaining period. Specifically, the release degree was about 25% in 5 s and it increased slowly to about 35% for the remaining 55 s. The hydrophobic side chain (i.e., methylene methyl sulfide) of the copolymer would be able to be anchored in between DOPE molecules through hydrophobic interaction while the hydrophilic HEA segments orienting toward aqueous bulk phase. This was thought to be a mechanism with which DOPE liposomes were stabilized by the copolymer. If the copolymer is exposed to an oxidative condition, the sulfide can be oxidized to become sulfoxide and sulfone (Figure 3) [29,30]. Upon the oxidation, the hydrophobic anchor was likely to become hydrophilic, it would hardly be kept to be anchored into the liposomal bilayers and the copolymers would be removed from the liposomal bilayers, leading to the disintegration of the liposomes and the triggered release of the liposomal content. In fact, the copolymer lost its interfacial activity after treated with H_2_O_2_ (Figure 4). However, the release degree was higher at the two highest H_2_O_2_ concentrations during the whole period of release experiment. Thus, the maximum release degree at the concentration of 1.0% was about 78.0% and when H_2_O_2_ concentration was 2.0%, the release degree was about 78.4%, almost the same. As H_2_O_2_ concentration was higher, the sulfide copolymer was likely to be oxidized more readily and DOPE liposomes would be destabilized more extensively, leading to higher release degree. The oxidation degree of the sulfide copolymer at the concentration of 1.0% seemed to be already high enough to disintegrate the liposomes completely. The H_2_O_2_ concentration-dependent release profiles of dye enveloped in liposome/F-P(HEA-AMS)(100/1) were like those of dye enveloped in liposome/F-P(HEA-AMS)(200/1). Liposome/F-P(HEA-AMS)(100/1) exhibited no significant release when the release medium was free of H_2_O_2_. But it showed a triggered release when the release medium contained the oxidizing agent. For example, the maximum release degree was about 0.5%, 60.6%, 79.3%, and 80.2%, respectively, when the concentration of the oxidizing agent was 0%, 0.5%, 1.0%, and 2.0%. As in case of liposome/F-P(HEA-AMS)(200/1), the release degree at the concentration of 1.0% was higher than that observed at the concentration of 0.5% and no significant difference was found between the release degree at the concentration of 1.0 % and that at 2.0%, suggesting that the oxidation at 1.0% was already high enough to induce the complete disintegration of the liposomes. At the concentration of 0.5%, liposome/F-P(HEA-AMS)(100/1) exhibited higher release degree than liposome/F-P(HEA-AMS)(200/1). Since the former liposomes incorporate more sulfide copolymer for a fixed amount of DOPE than the latter liposomes, they would be subjected more readily to the oxidation-induced destabilization. The H_2_O_2_ concentration-dependent release profiles of dye for liposome/F-P(HEA-AMS)(50/1) were quite different. The maximum release degree was about 0.6%, 77.2%, 83.2%, and 83.7%, when the H_2_O_2_ concentration was 0%, 0.5%, 1.0%, and 2.0%, respectively. The liposome/F-P(HEA-AMS)(50/1) showed higher release degree at the H_2_O_2_ concentration 0.5% than the other liposomes and there was no significant difference between the release degree at H_2_O_2_ concentrations of 0.5%, 1.0% and 2.0%. These indicated that liposome/F-P(HEA-AMS)(50/1) was the most sensitive to H_2_O_2_ among the liposomes tested. Since liposome/F-P(HEA-AMS)(50/1) was incorporating higher amount of the sulfide copolymer than the other two kinds of liposomes, the amount of copolymer which can undergo oxidation would also be greater and the liposome was likely to be more susceptible to destabilization in an oxidative condition. This could be a reason why liposome/F-P(HEA-AMS)(50/1) was more sensitive to the oxidizing agent than liposome/F-P(HEA-AMS)(200/1) and liposome/F-P(HEA-AMS)(100/1).

### 2.6. Observation of Interaction of Liposomes and Cancer Cell and Tumor Cells

**Figure 7:**
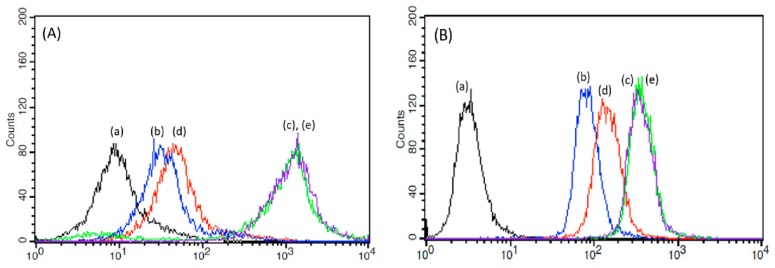
Fluorescence intensity distribution of KB cells (**A**) and LN229 cells (**B**) treated with free DOX (**a**), liposome/P(HEA-BMA)(100/1)/DOX (**b**), liposome/F-P(HEA-BMA)(100/1)/DOX (**c**), liposome/P(HEA-AMS)(100/1)/DOX (**d**), and liposome/F-P(HEA-AMS)(100/1)/DOX (**e**).

Figure 7 shows the fluorescence intensity distribution obtained using flow cytometry (FACS) of KB cells (a human cancer cell type originating from the cervix) (A) and LN229 cells (human glioma cell line) (B) treated with free DOX, liposome/P(HEA-BMA)(100/1)/DOX, liposome/F-P(HEA-BMA)(100/1)/DOX, liposome/P(HEA-AMS)(100/1)/DOX, and liposome/F-P(HEA-AMS)(100/1)/DOX. When the cells were treated with free DOX, the fluorescence intensity at the maximum cell count was about 10 and it was the lowest among the test samples tested. When the cells were treated with liposome/P(HEA-BMA)(100/1)/DOX, the fluorescence intensity at the maximum cell count was about 9000 and it was higher than that obtained with the cells treated with free DOX. This suggested that the liposomal DOX was internalized into the cells more efficiently than free DOX. It was reported that particulate matters including liposomes were translocated into the cells via particulate endocytosis and small solutes including DOX were taken up by the cells via pinocytosis [31,32]. Small solutes would also be able to be internalized into the cells via simple diffusion. Since DOX was loaded and localized in the liposomes (specific loading %: 1.82–2.39), the cellular uptake of liposomes seemed to transport DOX to the cells more effectively than the molecular level transport phenomena (i.e., pinocytosis). The cellular uptake would be a reason why the fluorescence intensity of the cells treated with liposome/P(HEA-BMA)(100/1)/DOX was stronger than that of the cells treated with free DOX. When the cells were treated with liposome/F-P(HEA-BMA)(100/1)/DOX, the fluorescence intensity at the maximum cell count was about 1.5 × 10^3^ and it was much stronger than that obtained with the cells treated with liposome/P(HEA-BMA)(100/1)/DOX. Folate-receptor was reported to be overexpressed on the surface of KB cells and LN229 cells [33,34,35,36]. Thus, the folate-conjugated liposome would readily be able to bind to the cancer cells and tumor cells through receptor-ligand interaction then the liposome would be likely to be internalized into the cells via receptor-mediated endocytosis. The strong fluorescence intensity of the cancer cells and tumor cells treated with the folate-conjugated liposomes could be ascribed to the receptor-mediated uptake. The cancer cells and the tumor cell treated with liposome/P(HEA-AMS)(100/1)/DOX and liposome/F-P(HEA-AMS)(100/1)/DOX showed similar fluorescence intensity distributions to the cancer cells and tumor cells treated with liposome/P(HEA-BMA)(100/1)/DOX and liposome/F-P(HEA-BMA)(100/1)/DOX, respectively. This indicates that the former liposomes were as potent as the latter ones in terms of their translocation into the cancer cells and tumor cells.

**Figure 8:**
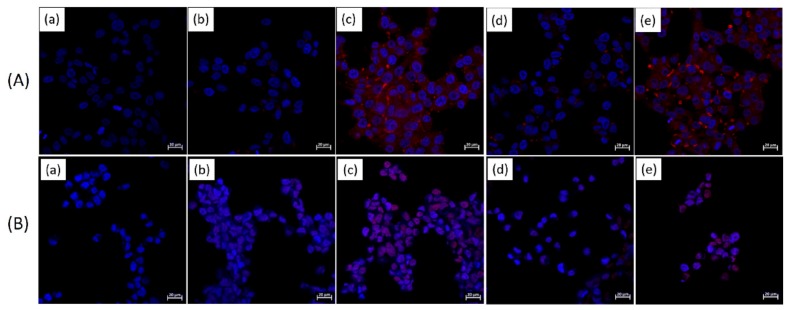
CLSM micrograph of KB cells (**A**) and LN229 cells (**B**) treated with free DOX (**a**), liposome/P(HEA-BMA)(100/1)/DOX (**b**), liposome/F-P(HEA-BMA)(100/1)/DOX (**c**), liposome/P(HEA-AMS)(100/1)/DOX (**d**), and liposome/F-P(HEA-AMS)(100/1)/DOX (**e**). In all cases, cells were also treated with DAPI to dye the nuclei.

Figure 8 shows the confocal laser scanning microscopy (CLSM) micrograph of KB cells (A) and LN229 cells (B) treated with free DOX, liposome/P(HEA-BMA)(100/1)/DOX, liposome/F-P(HEA-BMA)(100/1)/DOX, liposome/P(HEA-AMS)(100/1)/DOX, and liposome/F-P(HEA-AMS)(100/1)/DOX. When the cancer cells and tumor cells were treated with free DOX, DAPI-dyed nuclei (blue circular objects) were found with no trace of DOX (red color). When treated with liposome/P(HEA-BMA)(100/1)/DOX, weak DOX fluorescence appeared within and around the nuclei, suggesting that DOX was taken up by the cancer cells and tumor cells. As described above, the endocytosis of the liposome into the cells would promote the cellular internalization of DOX. When treated with liposome/F-P(HEA-BMA)(100/1)/DOX, DOX fluorescence was found within and near by the nuclei and the intensity was much stronger than that of the cells treated with the folate-free liposomes. Possibly due to the specific interaction of folate and its receptor, the endocytosis of the liposomes would be expedited, leading to a promoted cellular internalization of DOX. The cancer cells and tumor cells treated with liposome/P(HEA-AMS)(100/1)/DOX and liposome/F-P(HEA-AMS)(100/1)/DOX exhibited similar DOX fluorescence intensities to the cancer cells and tumor cells treated with liposome/P(HEA-BMA)(100/1)/DOX and liposome/F-P(HEA-BMA)(100/1)/DOX, respectively. This suggests that the feasibility of the cellular uptake of the former liposomes was as high as that of the latter ones.

### 2.7. Observation of In Vitro Anti-Cancer Cellular Efficacy

**Figure 9:**
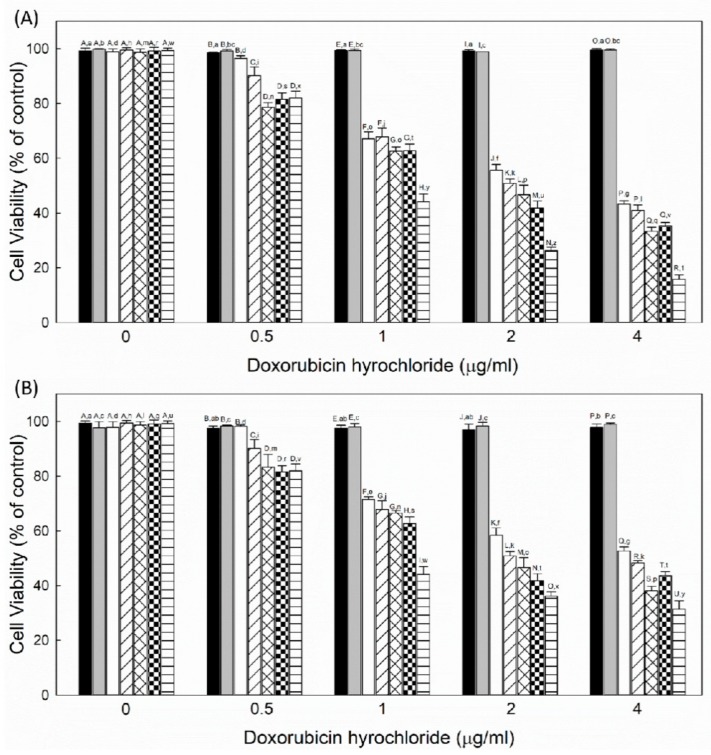
Viability of KB cells (**A**) and LN229 cells (**B**) treated with liposome/F-P(HEA-BMA)(100/1) (black bar), liposome/F-P(HEA-AMS)(100/1) (gray bar), free DOX (white bar), liposome/P(HEA-BMA) (100/1)/DOX (hashed bar), liposome/F-P(HEA-BMA)(100/1)/DOX (cross hatched bar), liposome/P(HEA-AMS)(100/1)/DOX (checkered bar), and liposome/F-P(HEA-AMS)(100/1)/DOX (striped bar).

Figure 9 shows the viability of KB cells (A) and LN229 cells (B) treated with liposome/F-P(HEA-BMA)(100/1), liposome/F-P(HEA-AMS)(100/1), free DOX, liposome/P(HEA-BMA)(100/1)/DOX, liposome/F-P(HEA-BMA)(100/1)/DOX, liposome/P(HEA-AMS)(100/1)/DOX, and liposome/F-P(HEA-AMS)(100/1)/DOX. When treated with liposomes without DOX (i.e., liposome/F-P(HEA-BMA)(100/1) and liposome/F-P(HEA-AMS)(100/1)), the viability of cancer cell and tumor cell fluctuated near 100% in the full range of concentration tested, suggesting that the liposomes exhibited no appreciable in vitro cytotoxicity under present experimental condition. The viability of the cancer cell and tumor cell treated with free DOX gradually decreased to about 43% in KB cells and 53% in LN229 cells when the concentration increased to 4 μg/mL. The viability of the cancer cell and tumor cell treated with liposome/P(HEA-BMA)(100/1)/DOX decreased to about 40% in KB cells and 48% in LN229 cells in the same concentration range and it was not significantly different from that of cells treated with free DOX. On the other hand, the viability of the cancer cell and tumor cell treated with liposome/F-P(HEA-BMA)(100/1)/DOX was significantly lower than that of cells treated with free DOX. For example, the viability of cells treated with the folate-conjugated liposome was about 33% in KB cells and 38% in LN229 cells at the DOX concentration of 4 μg/mL and it was lower than that of cells treated with DOX. The folate-conjugated liposomes would be targeted to the cancer cell and tumor cell through the specific interaction of folate and its receptor. In fact, FACS and CLSM revealed that liposome/F-P(HEA-BMA)(100/1) and liposome/F-P(HEA-AMS)(100/1) markedly promoted the delivery of DOX to the cancer cell and tumor cell. This could account for why the in vitro anti-cancer efficacy of liposome/F-P(HEA-BMA)(100/1)/DOX was significantly higher than that of free DOX. Meanwhile, the viability of cells treated with liposome/P(HEA-AMS)(100/1)/DOX was significantly lower than that of cells treated with liposome/P(HEA-BMA)(100/1)/DOX. For example, the viability of cells treated with the former liposome was about 35% in KB cells and 43% in LN229 cells at the DOX concentration of 4 μg/mL and it was lower than that of cells treated with the latter one. According to the results of FACS shown in Figure 7, liposome/P(HEA-AMS)(100/1) seemed to promote the intracellular delivery of DOX to almost the same degree as liposome/P(HEA-BMA)(100/1). However, liposome/P(HEA-AMS)(100/1) released its content in an oxidation responsive manner but liposome/P(HEA-BMA)(100/1) did not (Figure 6 and Figure S1). If the oxidation-sensitive liposomes are taken up by the cancer cell and tumor cell, P(HEA-AMS) chains would be oxidized and the liposomes would be able to destabilized, leading to the promoted release of their payload because the intracellular space of the cancer cell and tumor cell is in a high ROS level [37,38]. It is also known that oxygen free radicals are formed by DOX within the cancer cell and tumor cell and they peroxidize the cellular membrane lipids and exterminate the cancer cell and tumor cell [39,40,41]. The oxidation-sensitive liposomes would also be destabilized by the oxygen free radicals produced by DOX. This would be a reason why liposome/P(HEA-AMS)(100/1)/DOX was more efficacious than liposome/P(HEA-BMA)(100/1)/DOX in killing the cancer cell and tumor cell. The viability of cells treated with liposome/F-P(HEA-AMS)(100/1)/DOX markedly decreased to about 16% in KB cells, 31% in LN229 cells when the concentration increased to 4 μg/mL. Overall, LN229 cells showed less anticancer efficacy than KB cells, because the internalization was less than KB cells, as shown by FACS and CLSM (Figure 7 and Figure 8). Liposome/F-P(HEA-AMS)(100/1)/DOX exhibited the highest in vitro anti-cancer efficacy among the liposomes tested. According to the results of FACS and CLSM study shown, liposome/F-P(HEA-AMS)(100/1)/DOX seemed to be efficiently translocated into the cancer cell and tumor cell. In addition, the liposome could release its payload actively in an oxidative condition (Figure 6). These results could explain why liposome/F-P(HEA-AMS)(100/1)/DOX was so effective in killing the cancer cell and tumor cell.

## 3. Materials and Methods

### 3.1. Materials

1,2-Dioleoyl-sn-Glycero-3-Phosphoethanolamine (DOPE), 2-hydroxyethyl acrylate (HEA), butyl methacrylate (BMA), allyl methyl sulfide (AMS), folic acid (FA), dicyclohexylcarbodiimide (DCC), N,N-dimethylformamide (DMF), deoxycholic acid (DOC, sodium salt), calcein, Triton X-100, phosphate buffer solution (PB), phosphotungstic acid (PTA), thiazolyl blue tetrazolium bromide (MTT), fluoroshield^TM^ with DAPI, doxorubicin hydrochloride (DOX) and deuterium oxide (D_2_O) were purchased from Sigma-Aldrich Co. (St. Louis, MO, USA). Azobisisobutyronitrile (AIBN) was purchased from Junsei Chemical Co. (Tokyo, Japan). Phosphate buffered saline (PBS), RPMI 1640 medium (no folic acid), fetal bovine serum (FBS), penicillin-streptomycin, and trypsin-EDTA (0.25%) were purchased from Gibco^TM^ (Dublin, Ireland). Phosphorus pentoxide (P_2_O_5_) was purchased from Kanto Chemical Co., Inc. (Tokyo, Japan). Diethyl ether, dimethyl sulfoxide (DMSO), 4-dimethylaminopyridine (DMAP) and hydrogen peroxide (H_2_O_2_) were purchased from Daejung Chemical & Metals Co. Ltd. (Gyeonggi-do, Korea). Sephadex G-100 was provided by GE Healthcare (Uppsala, Sweden). Human nasopharyngeal epidermoid carcinoma cell line (KB) was purchased from the Korean cell line bank (Seoul, Korea). Human glioma cell line (LN229) was obtained from the American Type Culture Collection (ATCC, Manassas, VA, USA). All other reagents were of analytical grade.

### 3.2. Preparation of HEA Copolymers

Poly (HEA-*co*-AMS) (P(HEA-AMS)) and poly (HEA-*co*-BMA) (P(HEA-BMA)) were prepared by free radical reaction. In total, 5 g of HEA was dissolved in 50 mL of DMF in a 250 mL 3-neck round bottom flask. A hydrophobic comonomer (AMS and BMA) was dissolved in the HEA solution so that the HEA to comonomer molar ratio was 80:20. 40 mg of AIBN was added to the mixture monomer solution and the reaction mixture was purged with nitrogen gas for 30 min. The monomers were copolymerized at 75 °C with reflux for 12 h. After standing the flask containing reaction mixture until the temperature reached room temperature (20–23 °C), the copolymers were precipitated using diethyl ether as a non-solvent and they were purified by re-precipitation. The precipitates were separated by filtration and dried at room temperature.

### 3.3. Preparation of Folate-Conjugated HEA Copolymers

FA (0.320 g), DCC (1.534 g), and DMAP (0.904 g) were co-dissolved in 10 mL of DMSO contained in a 30 mL round bottom flask and the mixture solution was stirred using a magnetic bar at 30 °C for 2 h under dark condition and N_2_ atmosphere. In total, 0.856 g each of P(HEA-AMS) and P(HEA-BMA) was dissolved in 10 mL of DMSO, it was poured into FA solution, and the reaction mixture was stirred at 30 °C for 24 h. The byproduct (i.e., dicyclohexylurea) was removed by filtration, the filtrate was centrifuged at 12,000 rpm for 30 min. Then, the supernatant was dialyzed against distilled water using a dialysis tube (MWCO 1000) to remove impurities and unreacted FA. The dialyzed reaction product was freeze-dried for further use. Folate-conjugated P(HEA-AMS) and folate–conjugated P(HEA-BMA) were abbreviated to F-P(HEA-AMS) and F-P(HEA-BMA), respectively.

### 3.4. ^1^H NMR Spectroscopy

Residual solvent and water were removed by incubating copolymers with P_2_O_5_ in a vacuum incubator at 45 °C. Each of the dry copolymers was dissolved in D_2_O and the copolymer solution was put in a 10 mm NMR tube and sealed with a cap. The ^1^H NMR spectrum was obtained by scanning the copolymer solution on a NMR spectrophotometer (400 MHz, JNM-ECZ400S/L1, JEOL, Tokyo, Japan. installed in the Central Laboratory of Kangwon National University).

### 3.5. Treatment of Copolymers with H_2_O_2_

Each of copolymers (i.e., P(HEA-AMS) and P(HEA-BMA)) was dissolved in H_2_O_2_ solution (1% (v/v), in distilled water), the solutions were kept at room temperature (24 °C) for 10 min to oxidize the copolymers, and they were freeze-dried for further use. A copolymer treated with H_2_O_2_ solution was designated as H_2_O_2_ “copolymer name”.

### 3.6. Examination of Oxidation of Copolymers by XPS

The binding energy spectrum of P(HEA-AMS) and H_2_O_2_-P(HEA-AMS) were obtained by X-ray photoelectron spectroscopy (XPS, K Alpha+, Thermo Scientific, Basingstoke, UK) using a 180° double focusing hemispherical analyzer with 128-channel position sensitive detector. The reference value used to determine the binding energy of atomic electrons was the observed binding energy of C 1 s (284.8 eV). The copolymers were exposed to an achromatic Al K-alpha (1286.6 eV) X-ray source with the power of 72 W and the spectrophotometer was operated at room temperature (20–23 °C) and at a pressure of less than 5 × 10^−8^ mbar.

### 3.7. Measurement of Interfacial Tension 

The interfacial activity of H_2_O_2_-untreated and treated copolymers (i.e., P(HEA-AMS), H_2_O_2_-(HEA-AMS), P(HEA-BMA), and H_2_O_2_-P(HEA-BMA)) were examined by measuring the air/water interfacial tension of the copolymer solutions. Each of the copolymer solutions whose concentration was 0.01 mg/mL to 1.0 mg/mL was prepared by the continuous two times dilution of an aqueous copolymer solution (1.0 mg/mL). The interfacial tension was measured by a ring method on a tensiometer (DST 60, SEO, Suwon, Gyeonggi-do, Korea).

### 3.8. Preparation of DOPE Liposomes Stabilized with Copolymers

DOPE liposomes stabilized with F-P(HEA-BMA) and F-P(HEA-AMS) were prepared by a sonication and detergent removal method. The dry thin film of DOPE was prepared by removing the organic solvent from 2 mL of DOPE solution (10 mg/mL, in chloroform) contained in a 25 mL round bottom flask connected to a rotary evaporator operating at 150 rpm under reduced pressure. Variable amounts (0.02 mL, 0.04 mL, and 0.08 mL) of copolymer solution (5 mg/mL, in PB (10 mM, pH 7.4)), 0.09 mL of DOC solution (2% (w/v), in the same buffer solution), and 1 mL of calcein solution (100 mM, in the same buffer solution) were mixed in a 10 mL vial and the volume of the mixture solutions was made up to 2 mL by adding the buffer solution. When DOX-loaded liposomes were prepared, doxorubicin (DOX) solution (1 mL, 0.2% (w/v)) in PBS (10 mM, pH 7.4) was used instead of the calcein solution. In the mixture solutions, the concentration of copolymer was 0.05 mg/mL, 0.1 mg/mL or 0.2 mg/mL, and the concentration of DOC, calcein, and DOX were 0.09% (w/v), 50 mM, and 0.1%, respectively. 2 mL each of the mixture solutions was put in the round bottom flask containing dry DOPE film deposited on its inside wall and it was whirled by a hand to suspend the phospholipid in the mixture solution. The DOPE suspension was sonicated in a bath type of sonicator (VC 505, Sonic & Materials, Newtown, CT, USA) at room temperature for 20 min (pulse-on for 10 s and pulse-off for 10 s). DOPE liposomes containing calcein (or DOX) and copolymer were separated from unentrapped calcein (or DOX) and DOC by gel permeation chromatography using a Sephadex G-100 column. Liposome prepared from the mixture whose DOPE to copolymer mass ratio was x/y was termed as liposome/copolymer name (x/y) and the liposome containing DOX as liposome/copolymer name (x/y)/DOX.

### 3.9. Characterization of DOPE Liposomes Stabilized with Copolymers

The structure of DOPE liposomes stabilized with copolymers was investigated by transmission electron microscopy. In order to visualize the bilayer and the lamellar structure, the liposomes were negatively stained [42,43,44]. 100 μL of the liposome suspension was mixed with the same amount of phosphotungstic acid solution (2% (w/v)) and the mixture was stood at room temperature for 3 h for the staining of the liposomes. An aliquot amount of the stained liposome suspension was put on a formvar/copper-coated grid and it was dried overnight at room temperature. The replica was subjected to microscopy using a transmission electron microscope (TEM; LEO-912AB OMEGA, LEO, Germany; located in the Korea Basic Science Institute (KBSI), Kangwon, Chuncheon, Korea).

The fluorescence quenching of calcein enveloped in the liposome was determined using an Equation (1):

Quenching (%) = (F_t_ − F_i_) ÷ F_t_ × 100 (1)

where F_t_ is the fluorescence intensity after the liposomes were completely solubilized by Triton X-100 and F_i_ is the fluorescence intensity before solubilized [45,46]. The fluorescence intensity was measured at 514 nm with being excited at 495 nm using a fluorescence spectrophotometer (Hitachi F2500, Hitachi, Japan). The specific loading of DOX loaded in liposomes was determined as follows. DOX-loaded liposomes suspended in PBS (10 mM, pH 7.4) were solubilized using Triton X-100 and the fluorescence intensity of DOX in the solution was measured at 560 nm with being excited at 470 nm on a fluorescence spectrophotometer (Hitachi F2500, Hitachi, Japan). The amount of DOX was determined by reading the fluorescence intensity-corresponding concentration on a calibration curve. The calibration curve was set up by plotting the fluorescence intensity of DOX solutions (0–0.002 mg/mL, in PBS buffer (10 mM, pH 7.4)) versus the concentration. The specific loading % was calculated as the percent of the mass of DOX based on the mass of phospholipid. Dynamic light scattering method was used to measure the mean hydrodynamic diameter of DOPE liposomes stabilized with copolymers. The liposome suspensions were diluted with PB (10 mM, pH 7.4) to render the light scattering intensity to be 50–200 kcps, and the mean diameter was determined using a dynamic light scattering equipment (ZetaPlus 90, Brookhaven Instrument Co., Holtsville, NY, USA).

### 3.10. Observation of Oxidation-Sensitive Release

H_2_O_2_ (30%, *v*/*v*) was mixed with PB (10 mM, pH 7.4) so that the concentration was 0% (*v*/*v*), 0.5% (*v*/*v*), 1.0% (*v*/*v*), and 2.0% (*v*/*v*). 0.1 mL of liposome suspension was injected to 1.9 mL of H_2_O_2_ solution contained in a glass cuvette placed in the cuvette hold of a fluorescence spectrophotometer (Hitachi F2500, Hitachi, Japan). The fluorescence intensity was measured with time for 60 s. The excitation wavelength and emission one were 495 nm and 514 nm, respectively. The release % was calculated using the following Equation (2) [47,48]:

Release % =(F_t_ − F_i_) ÷ (F_f_ − F_i_) × 100 (2)

where F_i_ was the initial fluorescence intensity of the liposome suspension, F_f_ was the fluorescence intensity after the liposomes were completely solubilized by Triton X100, and F_t_ was the fluorescence intensity of the liposome suspension at a given time.

### 3.11. Observation of Interaction of Liposomes and Cancer Cell and Tumor Cell

KB cell and LN229 cell suspension (1 mL) were seeded in a 12-well plate (3 × 10^4^ cells/well) and cultured in a CO_2_ incubator at 37 °C for 24 h. The culture medium was decanted off, cells were washed with PBS (10 mM, pH 7.4), FBS-free RPMI 1640 culture medium (0.1 mL) and an equi-volumetric amount of each of test samples (free DOX solution, liposome/P(HEA-BMA)(100/1)/DOX suspension, liposome/F-P(HEA-BMA)(100/1)/DOX suspension, liposome/P(HEA-AMS)(100/1)/DOX suspension, and liposome/F-P(HEA-AMS)(100/1)/DOX suspension) was added to wells, and they were incubated for 5 h. Free DOX and unbound liposome were washed off by rinsing the cells with the buffer solution after the culture medium was removed. The cells were detached and harvested from the well wall by adding trypsin/EDTA solution to the wells. The cell suspensions were contained in Eppendorf tubes, they were centrifuged, and the supernatant was removed. After re-suspended in 500 μL of PBS (10 mM, pH 7.4) of 4 °C, the fluorescence intensity distribution of the cells was obtained using a flow cytometer (FACS, FACS Calibur, Becton Dickinson, Franklin Lakes, NJ, USA, in the Central Laboratory Center of Kangwon National University). The excitation and the emission wave length used to measure the fluorescence intensity were 470 and 560 nm, respectively. The interaction of DOX-loaded liposomes with the cancer cell and the tumor cell was also investigated by confocal laser scanning microscopy (CLSM). The cell culture condition in FACS study was used in CLSM study too. The cells treated with free DOX and DOX-loaded liposomes were treated with 200 μL of formaldehyde solution (2.5% (*v*/*v*)) for their structural fixation. After the cells were rinsed with PBS (10 mM, pH 7.4), the nuclei of cells were dyed using DAPI, free dye was removed by washing the cells with the buffer solution, and the CLSM micrographs were taken on a confocal laser scanning microscope (LSM 880 with Airyscan, Carl Zeiss, Oberkochen, Germany; installed in the Central Laboratory of Kangwon National University.

### 3.12. Observation of In Vitro Anti-Cancer Efficacy

The DOX concentration of liposome suspensions was adjusted to 5, 10, 20, and 40 μg/mL by diluting the suspensions with PBS (10 mM, pH 7.4). DOX-free liposomes suspensions (i.e., liposome/F-P(HEA-BMA)(100/1) and liposome/F-P(HEA-AMS)(100/1)) were used as positive controls. The liposome content of the control suspensions was made to be the same as that of the DOX-loaded liposome suspensions. DOX solution (in PBS (10 mM. pH 7.4)) was used as an additional positive control and PBS (10 mM, pH 7.4) as a negative control. Each well of a 96-well plate was seeded with 200 μL of cell (1 × 10^4^ cells/mL) and cultured at 37 °C for 24 h in a CO2 incubator. After the culture medium was taken off, RPMI free of FBS (180 μL) and each test sample (20 μL, DOX-loaded liposome suspensions, empty liposome suspensions, DOX solution, and blank PBS (10 mM, pH 7.4)) were put to each well, and incubated for 24 h in a CO_2_ incubator thermostated at 37 °C. The culture medium was taken off, the residual was washed off from the cell by rinsing them with PBS (10 mM, pH 7.4)), MTT reagent (20 μL, 5 mg/mL) was put to each well, and stored at the incubator for 4 h to induce the production of formazan. The supernatant was decanted off and formazan was dissolved out by adding DMSO (200 μL) to each well. The optical density of formazan solution was measured at 540 nm on a microplate reader (N10588, Thermo Fisher Scientific, Waltham, MA, USA). The percent of the optical density of formazan obtained from cells treated with a test sample or a control with respect to the optical density of formazan obtained from cells treated with the empty buffer solution was represented as cell viability.

## 4. Conclusions

Novel oxidation-sensitive liposomes were developed by stabilizing DOPE bilayers with F-P(HEA-AMS) and oxidation-insensitive liposomes were prepared as controls using F-P(HEA-BMA). According to the air/water interfacial tension measurement, the copolymers were surface-active and lost its surface-activity upon oxidation. Regardless of DOPE to copolymer mass ratio (200:1, 100:1, and 50:1), the release degree in 60 s of calcein enveloped in DOPE liposomes stabilized with F-P(HEA-BMA) slightly increased from a few to several % when H_2_O_2_ concentration increased from 0–2%. Meanwhile, the oxidizing agent concentration had a great effect on the release degree of dye enveloped in DOPE liposomes stabilized with F-P(HEA-AMS). The hydrophobic anchor (i.e., methylene methyl sulfide group) of the copolymer was likely to be oxidized to become hydrophilic and it would be desorbed from DOPE bilayers, leading to the disintegration of the liposomes. According to the results of FACS and CLSM study, the transport of DOX to cancer cell and tumor cell was markedly enhanced by liposomes stabilized with folate-conjugated copolymers (i.e., F-P(HEA-BMA) and F-P(HEA-AMS)), possibly because the liposomes were readily translocated into the cancer cell and the tumor cell via receptor-mediated endocytosis. The feasibility of the cellular uptake of liposomes with F-P(HEA-BMA) seemed to be almost the same as that of liposomes with F-P(HEA-AMS). However, the latter liposomes promoted the in vitro anti-cancer efficacy of DOX much more effectively than the former ones. This was possibly because liposomes with F-P(HEA-AMS) could release their payload sensitively in an oxidative condition.

## Supplementary Materials

The following are available online at https://www.mdpi.com/2072-6694/12/1/180/s1, Figure S1: H_2_O_2_ concentration-dependent release profiles of calcein.
