# Supplementary material for: Oxidation-Triggerable Liposome Incorporating Poly(Hydroxyethyl Acrylate-co-Allyl methyl sulfide) as an Anticancer Carrier of Doxorubicin
Source: Cancers (Basel). 2020 Jan 10;12(1):180. doi: 10.3390/cancers12010180 (PMC7017253; doi:10.3390/cancers12010180)
Supplement: Supplementary file 1 [file cancers-12-00180-s001.pdf]

Supplementary Material

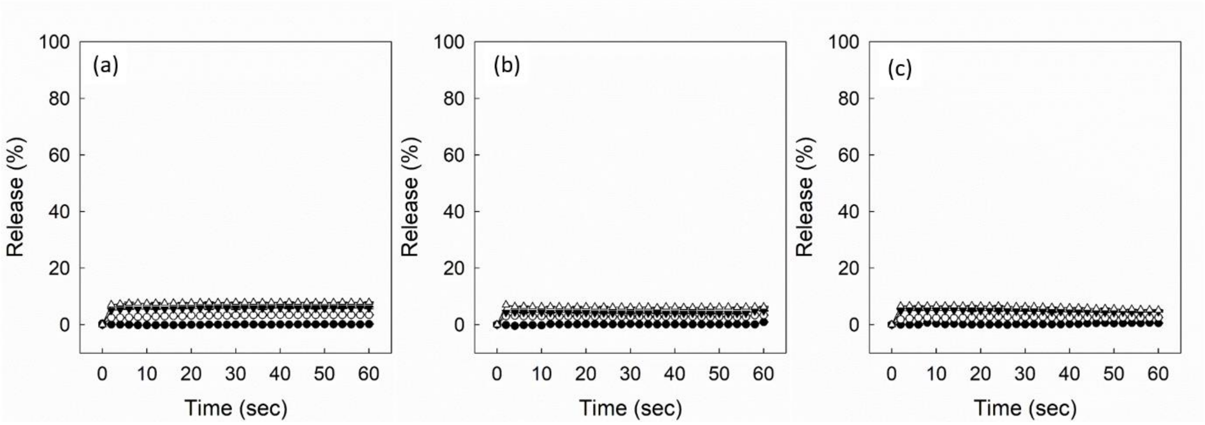

Figure S1. H_2O_2 concentration-dependent release profiles of calcein enveloped in liposome/P(HEA-BMA) (200/1) (**A**), liposome/P(HEA-BMA) (100/1) (**B**), and liposome/P(HEA- BMA) (50/1) (**C**) at 0 % (●), 0.5 % (○), 1.0 % (▼), and 2.0 %. (▽).
